# Favorable safety profile of moderate hypofractionated over normofractionated radiotherapy in breast cancer patients: a multicentric prospective real-life data farming analysis

**DOI:** 10.1186/s13014-022-02044-z

**Published:** 2022-04-20

**Authors:** Irfane Issoufaly, Claire Petit, Sébastien Guihard, Rémi Eugène, Loic Jung, Jean Baptiste Clavier, Stéphanie Servagi Vernat, Sara Bellefqih, Benjamin Verret, Naïma Bonnet, Éric Deutsch, Sofia Rivera

**Affiliations:** 1 Department of Radiotherapy, Gustave Roussy, Université Paris-Saclay, 94805 Villejuif, France; 2 Radiotherapy, Paul Strauss, Strasbourg, France; 3 Radiotherapy, Elekta, Paris, France; 4 Radiotherapy, Jean Godinot, Reims, France; 5 Unicancer Radiation and Oncology Group, Paris, France; 6 UMR 1030, Molecular Radiotherapy and Therapeutic Innovation, INSERM, 94805 Villejuif, France

**Keywords:** Breast cancer, Hypofractionation, Radiotherapy, Data-farming, Acute toxicity, Real-life data

## Abstract

**Background:**

Moderately hypofractionated whole-breast radiotherapy (HFRT) has proven to be as safe and efficient as normofractionated radiotherapy (NFRT) in randomized trials resulting in major changes in clinical practice. Toxicity rates observed in selected clinical trial patients may differ from those observed in unselected patients with possible comorbidities and frailty in real-life. This study aimed to examine the influence of HFRT versus NFRT on acute toxicity and identify risks factors of dermatitis in real-life patients.

**Materials and methods:**

Prospective data from breast cancer patients, treated with locoregional radiotherapy were collected between November 2015 and February 2020 in 3 comprehensive cancer centers. Through a systematic data-farming strategy, acute toxicity evaluation forms (CTCAEv4.0) were prospectively completed and extracted electronically. The results from each center were then anonymously merged into a single database for analysis. A Chi-2 test was used to compare HFRT and NFRT. Furthermore, risk factors of dermatitis were identified in a sub-study (622 patients) by multivariate logistic regression analysis.

**Results:**

In total, 3518 T0-4 N0-3 mostly M0 (85.8%) breast cancer patients with a median age of 60.7 (24–96 years old) were analyzed. Acute grade 2–3 dermatitis, grade 1–3 breast oedema, and grade 1–2 hyperpigmentation were less frequent with HFRT versus NFRT: respectively 8.9% versus 35.1% (Chi-2 = 373.7; *p* < 0.001), 29.0% versus 37.0% (Chi-2 = 23.1; *p* < 0.001) and 27.0% versus 55.8% (Chi-2 = 279.2; *p* < 0.001). Fewer patients experienced pain with HFRT versus NFRT: 33.4% versus 53.7% respectively (Chi-2 = 137.1; *p* < 0.001). Factors such as high BMI (OR = 2.30 [95% CI, 1.28–4.26], *p* < 0.01), large breast size (OR = 1.88 [95% CI, 1.07–3.28], *p* < 0.01) and lumpectomy over mastectomy (OR = 0.52 [95% CI, 0.27–0.97], *p* < 0.05) were associated with greater risk factors of grade 2–3 dermatitis in multivariate analysis regardless of NFRT or HFRT.

**Conclusion:**

The results of this study suggests that breast HFRT may be a better option even for patients with a high BMI or large breast size. Acute toxicity was low to mild, and lower with HFRT compared to NFRT. Results from real-life data were robust, and support the use of HFRT beyond randomized study populations. Long-term real-life data awaits further investigation.

## Introduction

Breast cancer is the most common cancer in women and the leading cause of cancer mortality [1]. Standard treatment includes adjuvant radiotherapy which improves local control, disease free survival and overall survival [2, 3].

Several phase III randomized studies have shown non-inferiority of moderate hypofractionated radiotherapy (HFRT) (39–42.5 Gy in 13 to 16 fractions of 2.67–3.2 Gy over 3–5 weeks) versus normofractionated radiotherapy (NFRT) (50 Gy in 25 fractions of 2 Gy over 5 weeks) in terms of overall survival, local relapse, recurrence-free survival, acute and late toxicity [4–7].

With a low α/β ratio, normal tissue toxicity mainly affected by doses per fraction, tends to be higher with HFRT than with NFRT. However, within the 10 years of follow up, no increase of acute or late toxicity has been observed in these studies [4, 8].

Moderate HFRT has been recognized as a standard of care for whole breast radiotherapy, resulting in major changes in clinical practice [9, 10].

Nevertheless, toxicity observed in randomized studies may differ from those observed in real-life patients. Patients with comorbidities, frailty or large tumors (i.e. T3 or T4) are likely to be excluded from randomized trials, which may have an impact on toxicity rates.

Studies evaluating the results of randomized clinical trials and real-life data on the same scientific question have shown discordant results [11–13]. Randomized clinical trials remain the gold standard for establishing guidelines, however real-life data are gaining significant attention over the last several years [14].

Even so, large-scale multicenter, prospective, standardized and pre-defined analysis of real-life data are still lacking in the field of breast radiation therapy.

Data-farming strategy is the process of using predesigned computational tools to “grow” data from structured models, which can then be easily electronically extracted, and statistically analyzed. The data from each treated patient “feeds” the system and contributes to “grow” the whole database facilitating a multicenter sharing process.

The main purpose of this study was to determine the influence of HFRT versus NFRT on acute toxicities, using a data-farming strategy, based on a structured, systematic, real-life evaluation form.

Moreover, risk factors for acute dermatitis were analyzed in a sub-study, based on retrospective data from one of the three participating centers.

## Materials and methods

### Data collection

Since November 2015, a common, standardized and structured acute toxicity evaluation form was implemented and systematically used in the MOSAIQ© software in three participating comprehensive cancer centers within the data farming strategy of UNITRAD (Unicancer group of Translational research and development in Radiation oncology). Evaluation forms were completed prospectively for all breast cancer patients treated with adjuvant radiotherapy by a radiation oncologist, weekly during radiotherapy, and during the last week of radiotherapy at the end of treatment consultation for both fraction schedules (week 3 or 5 from the beginning of HFRT or NFRT respectively).

In a sub-study, additional characteristics from all patients from one of the three participating centers were collected retrospectively for acute dermatitis risk factors including weight, height, body mass index (BMI), bra cup size before surgery, adjuvant or neoadjuvant chemotherapy and surgery type (lumpectomy or mastectomy).

### Data extraction

First, data from Mosaiq© evaluation forms were weekly and electronically extracted, to an SQL (Structured Query Language) database by Elekta Consulting SAS.

Then, data were anonymized and aggregated using an R programming code. Results from these extraction requests were shared by the principal investigator of each participating center under the umbrella of UNITRAD within a data transfer agreement. Data were computed in a single global anonymized database under excel format.

Extractions included clinical data (age, cTNM, pTNM, histology, estrogen receptor (ER), progesterone receptor (PR), HER2 status), radiotherapy data (prescribed dose, fractionation, treatment technique), acute toxicity evaluation forms (performance status, visual analog scale for peak and background pain from 0: no pain to 10: worse possible pain; with 1–5 being low pain and 6–10 intense pain, CTCAE V 4.0 grade for dermatitis, hyperpigmentation and breast oedema).

### Treatment regimens

External beam radiotherapy was delivered with a 3D conformal or an IMRT technique.

Regional nodal radiotherapy and tumor bed boost were delivered according to local and national guidelines as well as systemic therapy [15, 16]. Detailed information on which lymph node levels were irradiated were not available.

### Statistical analysis

Descriptive statistics were used for population and treatment characteristics and toxicity rates (number, percentage, minimum, maximum, inter-quartile range).

Analytic statistics were performed using the Chi-2 test, to compare the percentage of toxicity. Statistical tests were two sided, and *p* < 0.05 was considered statistically significant.

A sub-study was performed with a specific endpoint: development of acute radiation induced dermatitis (CTCAE Grade 2 or 3). Univariate analysis was carried out to assess the relationship between patient and clinical characteristics (BMI, bra cup size: A + B + C vs. ≥ D), type of surgery and the endpoint, separately for HFRT and NFRT. Variables with *p* value < 0.05 were tested in a multivariate logistic regression analysis including the type of radiotherapy, odds ratio with their 95% confidence interval (CI) were calculated.

All analyses were performed using RStudio software (version 1.2.5019).

### Data protection

This study, approved by the Institutional Review Board (No 2021-21) was in accordance with GDPR (General Data Protection Regulation) and with the standard of the CNIL (National Commission of Information and Liberty) “MR-004” [17].

An assessment of the study was performed by a Data Protection Officer (DPO) who implemented recommendations, including security measures to protect patient data and confidentiality.

Prior to the beginning of the trial, all patients were provided with a written informed consent by the principal investigator of each participating center. Out of 3519 patients, only 1 denied participation and was therefore not included in the analysis.

## Results

Between November 2015 and February 2020, data from 3518 patients were analyzed. The median age was 60.7 years (range 24–96 years). Among those, 1857 patients (52.8%) were between 50 and 70 years old. There were more elderly patients (> 70 years) in the HFRT group than in the NFRT group (44.7% vs 15.6% respectively). The majority of patients, 3412 (96.9%) had a performance status of 0–1. A total of 2510 (71.3%) patient tumors were ER+, 2171 (61.7%) were PR + and 377 (10.7%) HER2 overexpressed. Most patients had cT1-T2 n = 2662 (75.7%), cN0 n = 2148 (61.1%) with an invasive ductal carcinoma n = 2572 (73.1%) and only 108 (3.1%) were cM1 (Table 1).

**Table 1:** Patient and tumor characteristics

Characteristics	NFRT (n = 2394) n (%)	HFRT (n = 1124) n (%)	Total (n = 3518) n (%)
Age (years old)			
< 40	181 (7.6)	20 (1.8)	201 (5.7)
40–50	518 (21.6)	65 (5.8)	583 (16.6)
50–70	1321 (55.2)	536 (47.7)	1857 (52.8)
> 70	374 (15.6)	503 (44.7)	877 (24.9)
Performance status			
0	1767 (73.8)	771 (68.6)	2538 (72.1)
1	569 (23.8)	305 (27.1)	874 (24.8)
2–3–4	57 (2.4)	48 (4.3)	105 (3)
NA	1 (0.0)	0 (0.0)	1 (0.0)
Tumor estrogen receptor status			
ER−	368 (15.4)	91 (8.1)	459 (13.0)
ER+	1612 (67.3)	898 (79.9)	2510 (71.3)
NA	414 (17.3)	135 (12.0)	549 (15.6)
Tumor progesterone receptor status			
PR−	583 (24.3)	212 (18.9)	795 (22.6)
PR+	1395 (58.3)	776 (69.0)	2171 (61.7)
NA	416 (17.4)	136 (12.1)	552 (15.7)
Tumor HER2 status			
HER2−	1659 (69.3)	900 (80.0)	2559 (72.7)
HER2+	300 (12.5)	77 (6.9)	377 (10.7)
NA	435 (18.2)	147 (13.1)	582 (16.5)
Tumor grade			
1	335 (14.0)	279 (24.8)	614 (17.5)
2	873 (36.5)	522 (46.5)	1395 (39.7)
3	662 (27.6)	158 (14.0)	820 (23.3)
NA	524 (21.9)	165 (14.7)	689 (19.6)
cT			
T0	121 (5.1)	18 (1.6)	139 (4.0)
Tis	154 (6.4)	65 (5.8)	219 (6.2)
T1	1034 (43.2)	732 (65.1)	1766 (50.2)
T2	682 (28.5)	214 (19.0)	896 (25.5)
T3	182 (7.6)	35 (3.1)	217 (6.2)
T4	98 (4.1)	18 (1.6)	116 (3.3)
NA	123 (5.1)	43 (3.8)	165 (4.7)
cN			
N0	1243(51.9)	905(80.5)	2148 (61.1)
N1	686 (28.7)	90 (8.0)	776 (22.1)
N2	172 (7.2)	22 (2.0)	194 (5.5)
N3	89 (3.7)	18 (1.6)	107 (3)
NA	204 (8.5)	89 (7.9)	293 (8.3)
cM			
M0	2038 (85.1)	981 (87.3)	3019 (85.8)
M1	80 (3.4)	28 (2.5)	108 (3.1)
NA	276 (11.5)	116 (10.2)	391 (11.1)
Histology			
In situ carcinoma	153 (6.4)	79 (7.0)	228 (6.5)
Invasive ductal carcinoma	1748 (73.0)	821 (73.0)	2572 (73.1)
Invasive lobular carcinoma	255 (10.7)	121 (10.8)	376 (10.7)
Invasive ductal and lobular carcinoma	134 (5.6)	48 (4.3)	179 (5.1)
Other	22 (0.9)	20 (1.8)	36 (1.0)
NA	82 (3.4)	35 (3.1)	127 (3.6)

*HFRT* : hypofractionated radiotherapy, *NFRT : * normofractionated radiotherapy, *cTNM* : clinical classification for Tumor (T) Node (N) and Metastasis (M) AJCC 7th edition, *NA* : not available. Estrogen Receptor (ER) and Progesterone Receptor (PR) positive mean more than 10%. Values are number (percentage) unless otherwise specified

HFRT was used in 1124 patients (31.9%) with mostly 2.67 Gy per fraction n = 1079 (96.0%) and NFRT in 2394 patients (68.1%). Conformal 3D technique was used in 2878 patients (81.8%).

Among the patients who received HFRT, 508 (45.2%) received whole breast or chest wall radiotherapy without boost and 570 (50.7%) received whole breast radiotherapy with an additional boost of 4 × 2.5 Gy for 496 patients (44.1%), of 8 × 2 Gy for 60 patients (5.3%) and of 5 × 2.67 Gy for 14 patients (1.3%). Among the patients who received NFRT, 945 (39.5%) received whole breast radiotherapy without boost, and 1378 (57.5%) received whole breast radiotherapy with an additional boost of 8 × 2 Gy. Simultaneous integrated boost (SIB) was used for 39 patients (1.6%) (Table 2).

**Table 2:** Radiotherapy characteristics

Characteristics	NFRT (n = 2394) n (%)	HFRT (n = 1124) n (%)	Total (n = 3518) n (%)
Radiotherapy technique			
Conformal 3D	1878 (78.4)	1000 (89.0)	2878 (81.8)
IMRT	516 (21.6)	124 (11.0)	640 (18.2)
Total dose			
HFRT: 40.05 Gy (15 Fr × 2.67 Gy)	–	508 (45.2)	508 (14.4)
NFRT: 50 Gy (25 Fr × 2.0 Gy)	945 (39.5)	–	945 (26.9)
50.05 Gy (HFRT + Boost 4 Fr × 2.5 Gy)	–	496 (44.1)	496 (14.1)
53.4 Gy (HFRT + Boost 5 Fr × 2.67 Gy)	–	14 (1.3)	14 (0.4)
56.05 Gy (HFRT + Boost 8 Fr × 2.0 Gy)	–	60 (5.3)	60 (1.7)
66 Gy (NFRT + Boost 8 Fr × 2.0 Gy)	1378 (57.5)	–	1378 (39.2)
64.4 Gy (28 Fr × 1.8 (NFRT); 28 Fr × 2.3 Gy SIB)	18 (0.8)	–	18 (0.5)
68.32 Gy (28 Fr × 1.8 (NFRT); 28 Fr × 2.44 Gy SIB)	21 (0.9)	–	21 (0.6)
Other	32 (1.3)	46 (4.1)	78 (2.2)
Boost			
No	945 (39.5)	508 (45.2)	1453 (41.3)
Yes/sequential boost	1378 (57.5)	570 (50.7)	1948 (55.4)
Yes/SIB	39 (1.6)		39 (1.1)
Other	32 (1.4)	46 (4.1)	78 (2.2)
Dose per fraction (Gy)			
2	2394 (100)	–	2394 (68.1)
> 2.2 (equal to 2.67)	–	1124 (100) 1079 (96.0)	1124 (31.9) 1079 (30.7)

*Fr* : fraction, *IMRT :* intensity modulated radiotherapy, *HFRT* : hypofractionated radiotherapy, *NFRT* : normofractionated radiotherapy, *SIB :* simultaneous integrated boost. Values are number (percentage) unless otherwise specified

**Fig. 1:**
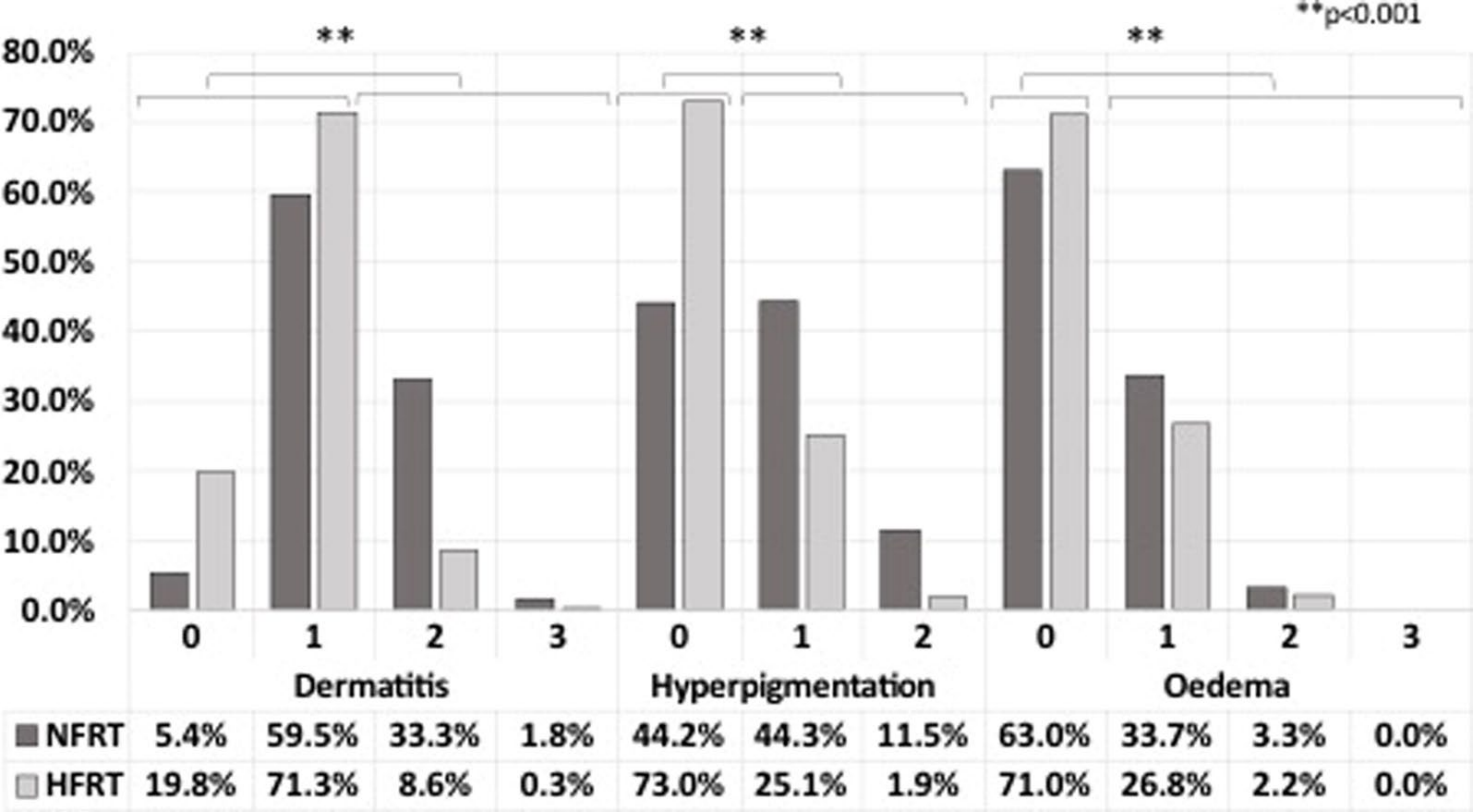
Acute cutaneous toxicities according to grade and fractionation.Histogram reporting the percentage of CTCAE V 4.0 grade 0–5 dermatitis, hyperpigmentation and breast oedema according to fractionation (normofractionated versus hypofractionated radiotherapy). *HFRT* : hypofractionated radiotherapy, *NFRT* : normofractionated radiotherapy. Histograms for toxicity grade 0–5 are, only presented when any of each grade was present. Acute toxicity evaluation was performed once a week and at the end of radiotherapy. Data were extracted from the evaluation form, filled in at the end of radiotherapy, which reported the maximum acute toxicity reached during treatment

Overall, radiation-related acute toxicity observed after HFRT or NFRT were low to mild and no grade 4 or 5 toxicities were reported. Dermatitis, breast oedema and hyperpigmentation were less frequent with HFRT, compared to NFRT. The proportion of patients who experienced toxicity was 8.9% versus 35.1% for dermatitis grade 2–3 (Chi-2 = 373.7; *p* < 0.001), 27.0% versus 55.8% for hyperpigmentation grade 1–2 (Chi-2 = 279.2; *p* < 0.001) and 29.0% versus 37.0% for breast oedema grade 1–3 (Chi-2 = 23.1; *p* < 0.001) respectively (Fig. 1).

**Fig. 2:**
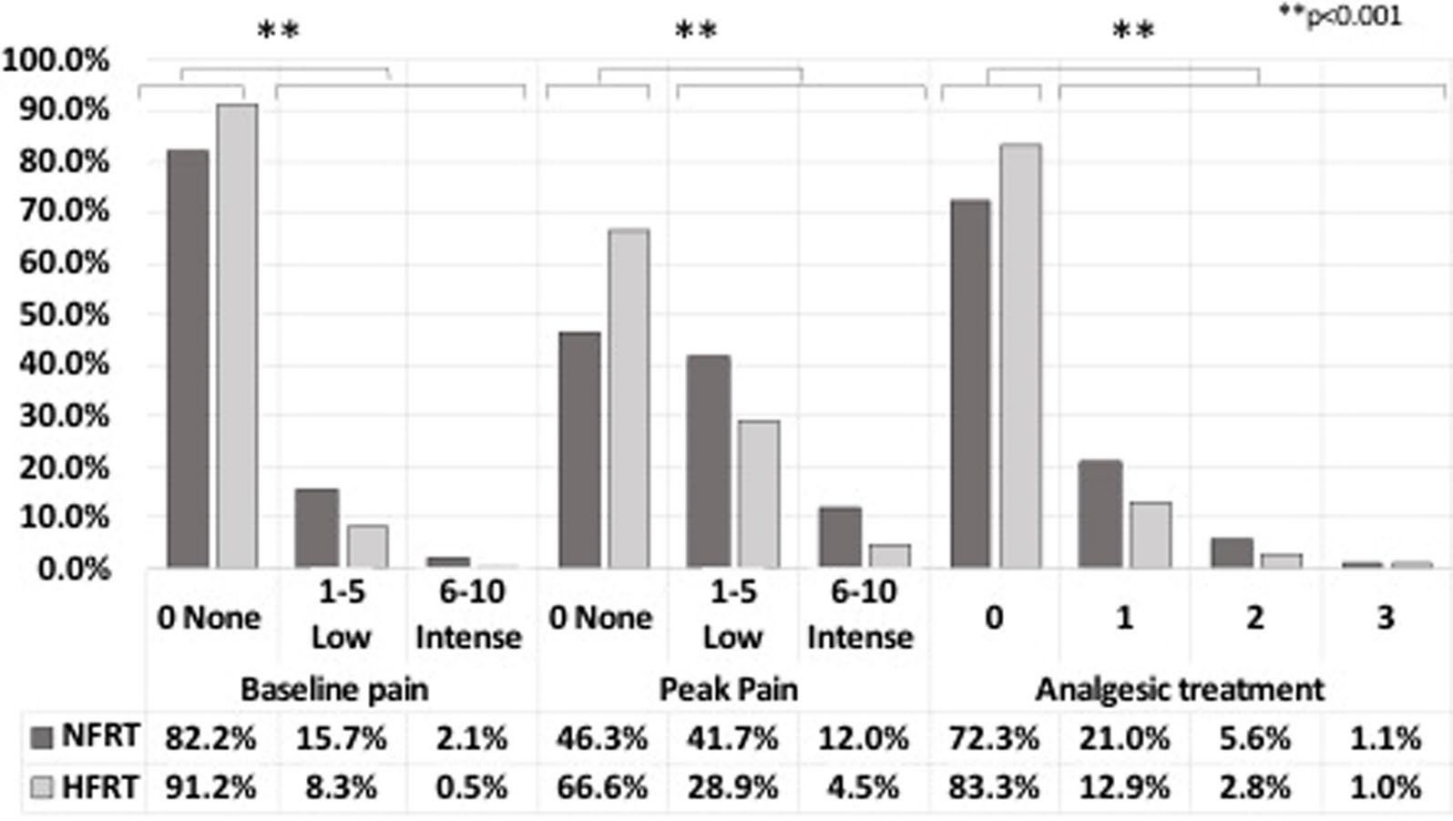
Pain and analgesic treatment according to grade and fractionation. Histogram reporting the percentage of patients with corresponding level of maximum pain and the need of analgesic treatment according to fractionation (normofractionated versus hypofractionated radiotherapy). *HFRT*: hypofractionated radiotherapy, *NFRT*: normofractionated radiotherapy. Analgesic treatment level was determined from the World Health Organization’s pain killers ladder (0 = none; 1 = Non opioid analgesic; 2 = Weak opioid; 3 = Strong opioid). Acute toxicity evaluation was performed once a week during radiotherapy and at the end of radiotherapy. Data were extracted from the evaluation form, filled in at the end of radiotherapy, which reported the maximum acute toxicity reached during treatment

Patients reported significantly less pain with HFRT compared to NFRT. Low or intense background pain was 8.8% with HFRT versus 17.8% with NFRT (Chi-2 = 53.7; *p* < 0.001) and low or intense peak pain was 33.4% with HFRT versus 53.7% with NFRT (Chi-2 = 137.1; *p* < 0.001). Consistently, the need for analgesic treatment was less frequent for patients undergoing HFRT compared to NFRT: 16.7% versus 27.7% respectively (Chi-2 = 52.7; *p* < 0.001) (Fig. 2).

No significant difference was reported regarding dermatitis between N0 and N + patients regardless of the fractionation schedule (Chi2 = 0.24; *p* = 0.62), however lower grade 2–3 dermatitis was noted in the HFRT group compared to the NFRT in N0 group (Chi2 = 26.49; *p* < 0.001) and in N + group (Chi2 = 14.35; *p* < 0.001).

An additional subgroup analysis was performed on 622 patients from one of the participating centers, to identify possible risk factors of developing radiation-related acute dermatitis, according to fractionation. Grade 2–3 dermatitis distribution related to risk factors and to fractionation is reported in Table 3.

**Table 3:** Grade 2–3 dermatitis according to risks factors and fractionation

Dermatitis grade 2–3	Total (n = 622)	NFRT (n = 317)	*p* value univariate NFRT	HFRT (n = 305)	*p* value univariate HFRT	*p* value multivariate on all patients^a^	Odds ratio multivariate on all patients^a^ [CI 95%]
Body mass index							
< 25 kg/m^2^	25/280 (8.9)	19/130 (14.6)	< 0.001	6/150 (4.0)	0.17	0.007	2.30 [1.28–4.26]
≥ 25 kg/m^2^	85/339 (25.1)	73/185 (39.5)		12/154 (7.8)			
NA	3 (0.5)	1/2		0/1			
Bra cup size							
A–C	47/351 (13.4)	39/172 (22.7)	< 0.001	8/179 (4.5)	0.17	0.03	1.88 [1.07–3.28]
D–J	45/154 (29.2)	39/89 (43.8)		6/65 (9.2)			
NA	117 (18.8)	15/56		4/61			
Chemotherapy							
No	35/295 (11.9)	25/74 (33.8)	0.34	10/221 (4.5)	0.11	0.59	0.84 [0.45–1.57]
Yes	76/327 (23.2)	68/243 (28.0)		8/84 (9.5)			
Type of surgery							
Lumpectomy	62/412 (15.0)	45/142 (31.7)	0.02	17/270 (6.3)	0.98	0.04	0.52 [0.27–0.97]
Mastectomy	28/177 (15.8)	28/145 (19.3)		0/32 (0.0)			
Other	33 (5.3)	20/30		1/3			

Values are number (percentage) unless otherwise specified

*HFRT* : hypofractionated radiotherapy, *NFRT* : normofractionated radiotherapy, *CI* : confidence interval, *NA* : not available

^a^With addition of the covariate type of radiotherapy (HFRT or NFRT)

For patients treated with HFRT, high BMI (≥ 25 kg/m^2^), large bra cup size (over C cup), chemotherapy and the type of surgery, were not found to be significantly associated with grade 2–3 dermatitis in univariate analysis (*p* = 0.17 for BMI and bra cup size, *p* = 0.11 for chemotherapy and *p* = 0.98 for type of surgery).

For patients treated with NFRT, high BMI, large bra cup size and lumpectomy were found to be significantly associated with grade 2–3 dermatitis in univariate analysis (*p* < 0.001 for BMI and bra cup size, *p* < 0.05 for surgery type).

In multivariate analysis, with the addition of the fractionation schedule covariable, BMI (OR = 2.30 [95% CI, 1.28–4.26], *p* < 0.01), bra cup size (OR = 1.88 [95% CI, 1.07–3.28], *p* < 0.01) and lumpectomy (OR = 0.52 [95% CI, 0.27–0.97], *p* < 0.05) were found to be statistically significant risk factors for grade 2–3 acute dermatitis. On the contrary, chemotherapy was not found as a statistically significant risk factor to developing acute grade 2–3 dermatitis (OR = 0.84 [95% CI, 0.45–1.57], *p* = 0.59).

Moreover, the percentage of grade 2–3 dermatitis remained lower with HFRT than NFRT in each subgroup, as shown in Table 3.

## Discussion

Moderate HFRT remains the standard of care for whole breast radiotherapy based on phase III randomized studies showing non inferiority in terms of efficacy and safety [4, 7, 8].

Our study focuses on the evaluation of acute toxicity in real-life patients treated with whole breast or chest wall radiotherapy who may not fulfill all clinical trial inclusion criteria including patients with T3, N + or patients with large breasts who were excluded from the Ontario trial [4].

Results of this systematic, large-scale, multicentre prospective data collection, showed that both HFRT and NFRT had limited and acceptable acute toxicity in real-life patients. HFRT was associated with a significantly reduced risk of developing dermatitis, breast oedema, hyperpigmentation or pain.

In our series of 3518 patients, grade 2–3 dermatitis was observed in 8.9% of patients who received HFRT versus 35.1% of patients who received NFRT. These findings were consistent with the result from a sub study of the Ontario trial, including 161 of the 1234 randomized patients showing lower acute skin toxicity in the HFRT arm (12% for HFRT vs 38% for NFRT), which correlated with an overall increased quality of life [18].

Few studies have reported pain assessment in this context. We found 33.4% of patients experienced low or intense peak pain with HFRT versus 53.7% with NFRT. Consistently, a randomized series of 287 patients comparing HFRT versus NFRT for whole breast followed by tumor bed boost, also showed significantly less, low or intense maximum acute breast pain with HFRT (55% vs 74%. *p* = 0.001) [19].

A previous prospective population based study had included 2309 patients. All received adjuvant HFRT or NFRT to the whole breast exclusively after breast conserving surgery [20]. Similar results were found in our series including patients with a mastectomy and larger tumor; HFRT was associated with reduced acute pain compared to NFRT.

To our knowledge, this study is the largest prospective study on unselected real-life patients, including whole breast or chest wall radiotherapy, with or without tumor bed boost irradiation, and supports the safety of using HFRT in daily practice, regarding acute toxicities.

In all participating centers, patients with N + disease were systematically offered locoregional radiotherapy and locoregional radiotherapy was not recommended for N0 patients. Therefore N + patients who represented 30.6% of all patients could be a good approximation of patients treated with locoregional radiotherapy. There was no significant difference reported regarding dermatitis between N0 and N + patients regardless of the fractionation schedule. However lower grades 2–3 dermatitis were reported with HFRT than NFRT in both N0 and N + group.

The reported obstacles affecting the implementation of HFRT, include concerns regarding applicability of published trials to patients with higher toxicity risk factors [21, 22]. Some factors such as normofractionation, large breast size and high BMI are known to be associated with risks of developing acute skin toxicity [23] resulting in higher rates of acute adverse events, and worse cosmetic outcomes after adjuvant breast radiotherapy [24]. Our results showed in univariate analysis that high BMI and large bra cup size are not associated with more toxicity for HFRT but in multivariate analysis, independently from fractionation, obesity (BMI ≥ 25 kg/m^2^), large breast size (bra cup > C), and lumpectomy over mastectomy, were associated with a higher risk of grade 2–3 dermatitis. However it is known in literature that tumor bed boost is a major factor influencing acute toxicity [25] and lumpectomy as a risk factor of acute toxicity has to be balanced with the use of tumor bed boost, which influences the total dose received by patients and therefore the toxicity. Indeed, other studies focusing on patients treated with HFRT, with or without tumor bed boost, report more acute and late toxicity for patients treated with a tumor bed boost or a skin treated area receiving more than 20 Gy or > 400 cm^2^ [26, 27]. Interestingly the percentage of grade 2–3 dermatitis with HFRT remains lower than with NFRT in patients with every risk factors for toxicity. Our reassuring data therefore suggests that these risk factors should no longer be seen as obstacles to the use of HFRT.

The main strength of our study was to further support randomized trial results, regarding acute safety of HFRT. This study also showed how the use of prospective, structured, multicenter data extraction from a large number of unselected real-life patients, could help to monitor the implementation of a new standard, and may provide additional help for physicians to feel more comfortable offering HFRT. Such data extraction could also be used to provide information on the compliance to clinical standards, and provides relevant metrics for the monitoring of treatment quality, a cornerstone of clinical practice change.

This study had some limitations. First, this was an observational study differing from randomized trials by the use of real-life heterogeneous populations introducing possible selection bias. However, this method of evaluation could be implemented at a national and an international scale to support and monitor implementation of new standard in addition to randomized trials. Second, patients were not assessed with a systematic standardized evaluation form after the end of radiotherapy; therefore, these data were not included in the analysis, which may underestimate the maximum acute toxicity. This underestimation should be the same for both HFRT and NFRT as Arsenault et al. had shown a toxicity peak occurrence 1 week after the end of radiotherapy in both schedules [18]. Third, the retrospective sub-study for dermatitis risk factors was only performed on data from a single center due to limitations to additional data access. Nevertheless the entire population (622 patients) of this center contributed to the sub-study.

More recently, the randomized phase III Fast-Forward trial went further into hypofractionation, showing non-inferiority of 26 Gy in 5 fractions of 5.2 Gy over 1 week versus 40 Gy in 15 fractions over 3 weeks, in terms of local tumor control and safety at 5 years for early-stage breast cancer [28, 29].

Some centers could be reluctant to implement such a highly hypofractionated treatment in practice. Moving towards 1-week hypofractionation may also be safe as HFRT showed fewer early toxicities.

The prospective use of structured evaluation forms in daily workflow would however, allow continuous monitoring of patients toxicities, and could be of major interest to alert physicians in case of over-toxicity. Especially in the current context of the coronavirus disease pandemic, where a Fast Forward schedule has been rapidly endorsed by international guidelines, to restrict exposure of health-care professionals and patients to the virus, the prospective use of such structured evaluation forms could be even more important [30].

## Conclusion

Adjuvant breast HFRT raises no concerns in large-scale multicenter prospectively assessed real-life data from unselected breast cancer patients. Acute toxicity were low to mild, and lower with HFRT compared to NFRT regarding dermatitis, breast oedema, hyperpigmentation and pain.

Our real-life data study provides reassurance on the applicability of the results from HFRT randomized studies, and identifies risk factors of acute skin radiation related toxicity such as higher body mass index and larger breast size, which seemed reduced by using HFRT over NFRT. Further monitoring and continuous analysis of real-life acute and late toxicity in breast cancer radiotherapy is planned within the French UNITRAD network, which may assist in safe and confident harmonization, and change in practice.

## Data Availability

The datasets used and/or analyzed during the current study are stored in an institutional repository and will be shared upon reasonable request to the corresponding author.

## Abbreviations

BMI: Body mass index
CI: Confidence interval
CNIL: National Commission of Information and Liberty
CTCAE V 4.0: Common terminology criteria for adverse events version 4.0
DPO: Data Protection Officer
ER: Estrogen receptor
FR: Fraction
GDPR: General Data Protection Regulation
HFRT: Hypofractionated radiotherapy
IMRT: Intensity Modulated Radiotherapy
NFRT: Normofractionated radiotherapy
OR: Odds ratio
PR: Progesterone receptor
PS: Performance status
SEQ: Sequential boost
SIB: Simultaneous integrated boost
SQL: Structured Query Language
UNITRAD: Unicancer group of Translational research and development in Radiation oncology
